# Hypothesis of K^+^-Recycling Defect Is Not a Primary Deafness Mechanism for Cx26 (*GJB2*) Deficiency

**DOI:** 10.3389/fnmol.2017.00162

**Published:** 2017-05-26

**Authors:** Hong-Bo Zhao

**Affiliations:** Department of Otolaryngology, University of Kentucky Medical CenterLexington, KY, United States

**Keywords:** potassium recycling, deafness mechanism, connexin, gap junction, nonsyndromic hearing loss, cochlear development, miRNA, inner ear

## Abstract

K^+^-recycling defect is a long-standing hypothesis for deafness mechanism of Connexin26 (Cx26, *GJB2*) mutations, which cause the most common hereditary deafness and are responsible for >50% of nonsyndromic hearing loss. The hypothesis states that Cx26 deficiency may disrupt inner ear gap junctions and compromise sinking and recycling of expelled K^+^ ions after hair cell excitation, causing accumulation of K^+^-ions in the extracellular space around hair cells producing K^+^-toxicity, which eventually induces hair cell degeneration and hearing loss. However, this hypothesis has never been directly evidenced, even though it has been widely referred to. Recently, more and more experiments demonstrate that this hypothesis may not be a deafness mechanism underlying Cx26 deficiency. In this review article, we summarized recent advances on the K^+^-recycling and mechanisms underlying Cx26 deficiency induced hearing loss. The mechanisms underlying K^+^-sinking, which is the first step for K^+^-recycling in the cochlea, and Cx26 deficiency induced cochlear developmental disorders, which are responsible for Cx26 deficiency induced congenital deafness and associated with disruption of permeability of inner ear gap junctional channels to miRNAs, are also summarized and discussed.

## Introduction

Connexin26 (Cx26, *GJB2*) gene mutations are responsible for >50% of nonsyndromic hearing loss, causing either congenital deafness or late-onset progressive hearing loss (Zhao et al., 2006; del Castillo and del Castillo, 2011; Chan and Chang, 2014). Cochlear implants can restore hearing function of patients with Cx26 mutants, indicating major pathology of deafness in the cochlea. Several deafness mechanisms have been proposed, such as disruption of K^+^-recycling in the cochlea to cause cell degeneration and deafness (Santos-Sacchi and Dallos, 1983; Kikuchi et al., 1995; Zhao et al., 2006) and elimination of IP_3_-Ca^++^ wave spreading in the cochlear sensory epithelium (Beltramello et al., 2005). In particular, the hypothesis of K^+^-recycling defect has been long-term considered as the deafness mechanism of Cx26 deficiency and widely referred to. However, recent studies demonstrated that K^+^-recycling hypothesis may not be a deafness mechanism of Cx26 deficiency. In this review article, we will summarize recent advances on the studies of K^+^-recycling and Cx26 deficiency deafness mechanisms. Other information, such as gap junctional function in the cochlea, connexin deafness mutations and phenotypes, and deficiency-induced pathological changes in the cochlea, has been summarized extensively by previous reviews (e.g., Zhao et al., 2006; del Castillo and del Castillo, 2011; Chan and Chang, 2014; Wingard and Zhao, 2015).

### K^+^-Recycling in the Cochlea and Hypothesized Mechanism for Cx26 Deficiency Induced Hearing Loss

**Figure 1:**
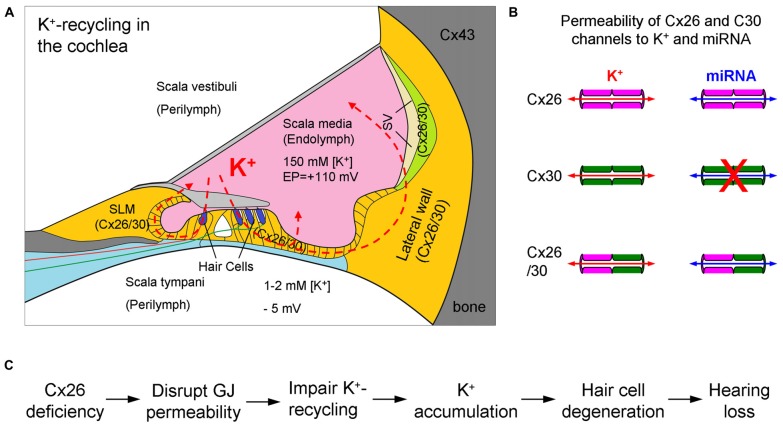
**K^+^-recycling in the cochlea and hypothesized deafness mechanism of Cx26 deficiency. (A)** Cochlear structure and K^+^-recycling pathways in the cochlea. Cx26 and Cx30 colocalized in most cochlear tissues and cells but not in hair cells. SLM, spiral limbus; SV, stria vascularis. Modified from Forge et al. (2003), Zhao and Yu (2006) and Liu and Zhao (2008). **(B)** Permeability of Cx26 and Cx30 gap junctional channels to ions and small molecules. Cx30 channels are impermeability to negative charged molecules, such as miRNAs. Based on Yum et al. (2010) and Zong et al. (2016). **(C)** The hypothesized K^+^-recycling defect as a mechanism for Cx26 deficiency induced hearing loss. GJ, gap junction.

**Figure 2:**
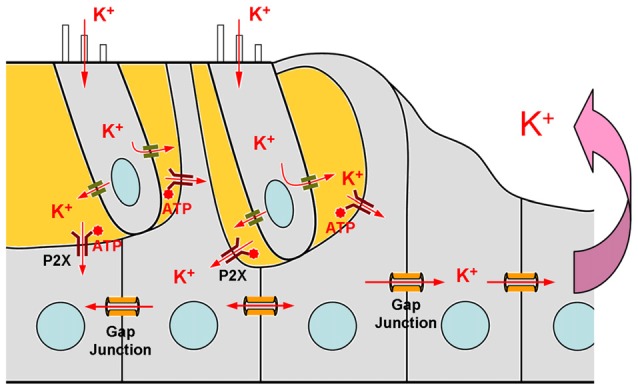
**Schematic drawing of the mechanism of ATP-P2X purinergic receptor-depended K^+^-sinking in the cochlear supporting cells.** Based on Zhu and Zhao (2010).

The cochlea is the auditory sensory organ, composed of three fluid-filled compartments, scala tympani (ST), scala media (SM) and scala vestibuli (SV). The ST and SV are filled with perilymph which is similar to the extracellular fluid with a high concentration of Na^+^ and low concentration of K^+^, whereas the SM is filled with endolymph which is similar to intracellular fluid with a low concentration of Na^+^ and high concentration of K^+^ (Figure 1A). The endolymph in the SM also possesses a high positive endocochlear potential (EP, +110–120 mV), which drives K^+^-ions in the endolymph passing through the mechano-transduction channels at hair cell’s hair bundles during acoustic simulation to produce auditory receptor current and potential, i.e., cochlear microphonics (CM). Influx K^+^ ions are then expelled out to the extracellular space through the lateral wall, which locates in the perilymph at the ST, to restore cell polarization. To avoid K^+^-toxicity and maintain hair cell function, the expelled K^+^ round hair cells needs to be removed. The K^+^-recycling hypothesis states that the expelled K^+^ ions are sunken by neighboring supporting cells and transported back to the endolymph via gap junction-mediated intracellular pathway between cells (Figures 1, 2).

This gap junction-mediated K^+^-recycling mechanism has been proposed since inner ear gap junctions were found about 35 years ago (Santos-Sacchi and Dallos, 1983; Kikuchi et al., 1995). After Cx26 mutations were found to be associated with hearing loss (Kelsell et al., 1997), this hypothesis was further linked to the mechanism of Cx26 mutations induced hearing loss (Figure 1C), since Cx26 is a predominant isoform in the cochlea (Kikuchi et al., 1995; Lautermann et al., 1998; Forge et al., 2003; Zhao and Yu, 2006; Liu and Zhao, 2008). That is, Cx26 mutations may impair inner ear gap junctions and disrupt the gap junction-mediated K^+^-recycling pathway in the cochlea, thereby causing K^+^ accumulation around hair cells producing toxicity, which eventually leads to hair cell degeneration and hearing loss (Figure 1C). Undoubtedly, K^+^-sinking and recycling in the cochlea are important and required for maintaining normal hair cell function and hearing. However, as a deafness mechanism of Cx26 deficiency, it lacks direct evidence, even though it has been widely referred to.

### Cx26 Deficiency Does Not Disrupt Inner Ear Gap Junctional Permeability and K^+^-Recycling Defect Hypothesis Is Not a Deafness Mechanism of Cx26 Deficiency

Recently, more and more experiments demonstrated that this hypothesized K^+^-recycling defect mechanism may not be a deafness mechanism of Cx26 deficiency. As described above, the hypothesis of disruption of K^+^-recycling by Cx26 deficiency causing hearing loss includes the following key-steps (Figure 1C): (I) Cx26 deficiency impairs cochlear gap junction permeability, thereby disrupting K^+^-transport between cochlear supporting cells. (II) Disruption of K^+^-recycling pathways leads to K^+^-accumulation around hair cells producing toxicity inducing hair cell degeneration. (III) Hair cell degeneration eventually causes hearing loss.

However, recent studies demonstrated that inner ear gap junctions in Cx26 knockout (KO) mice still remain permeability due to existence of co-expressed Cx30 (Zhu et al., 2015b). It has been found that gap junctions between cochlear supporting cells in Cx26 KO mice still retain good permeability to fluorescent dye ethidium bromide (EB; Zhu et al., 2015b), which molecular weight is 314 Da. This data suggests that inner ear gap junctions in Cx26 KO mice are still permeable to ions, including K^+^ ions.

Moreover, although Cx26 KO can induce congenital deafness and hair cell degeneration (Cohen-Salmon et al., 2002; Sun et al., 2009; Wang et al., 2009), hearing loss in Cx26 KO mice occurs in advance of hair cell degeneration (Liang et al., 2012), rather than behind the cell degeneration as assumed by the K^+^-recycling defect hypothesis. Also, deafness occurs in whole-frequency region (Liang et al., 2012), while the cell degeneration in Cx26 KO mice mainly locates at the middle and high frequency regions (Sun et al., 2009; Liang et al., 2012). These data further indicate that hair cell degeneration is not a primary cause for Cx26 deficiency induced hearing loss.

In addition, recent studies also showed that if Cx26 is conditionally deleted after postnatal day 4–5 (P4–5), mice demonstrated a progressive, late-onset hearing loss and had no apparent hair cell degeneration (Chen et al., 2014; Zhu et al., 2015a). Thus, Cx26 deficiency can cause hearing loss even without hair cell degeneration. Taken together, these findings indicate that the hypothesis of K^+^-recycling defect could not be a deafness mechanism for Cx26 deficiency induced hearing loss.

### K^+^-Sinking in the Cochlear Supporting Cells

K^+^-sinking or re-entering into the supporting cells is the first step for K^+^-recycling (Figure 2). However, it has been found that K^+^-sinking in the cochlear supporting cells is dependent on ATP-P2X receptors rather than Cx26 expression (Zhu and Zhao, 2010). ATP is an intracellular energy source. In the extracellular space, ATP can also act as a signaling molecule to activate purinergic (P2) receptors at the rest membrane potential evoking inward currents. P2 receptors have two subtypes: P2X ATP-gated ionotropic receptors and P2Y G-protein coupled metabotropic receptors. It has been found that the physiological level of ATP can activate P2X purinergic receptors producing inward currents in the cochlear supporting cells at normal physiological rest membrane potential (Zhu and Zhao, 2010). The inward current is linearly increased with extracellular K^+^ concentration increased, indicating that this inward current is mainly carried by K^+^ ions. Without ATP, no inward K current is visible in the cochlear supporting cells for high-concentration of extracellular K^+^ challenge (Zhu and Zhao, 2010). Interestingly, P2X2, which is a predominant P2X isoform in the cochlea, mutations can also induce nonsyndromic hearing loss (Yan et al., 2013; Faletra et al., 2014). These data demonstrate that K^+^-sinking is important for hair cell and hearing function. However, so far, there is no evidence indicating that this K^+^-sinking process is relative to Cx26 expression and that Cx26 deficiency can impair this K^+^-sinking process.

### Cx26 Deficiency Deafness Mechanisms

In the clinical, Cx26 mutations can cause both congenital deafness and late-onset hearing loss (del Castillo and del Castillo, 2011; Chan and Chang, 2014), also implicating that Cx26 deficiency induced hearing loss may have different underlying deafness mechanisms rather than a unique deafness mechanism as assumed by K^+^-recycling defect hypothesis.

For congenital deafness, Cx26 deficient mouse models show cochlear developmental disorders, hair cell degeneration and EP reduction (Cohen-Salmon et al., 2002; Sun et al., 2009; Wang et al., 2009; Liang et al., 2012; Chen et al., 2014). As mentioned above, hair cell degeneration is not a primary cause for Cx26 deficiency induced hearing loss, since deafness appears in whole-frequency range and occurs in ahead of hair cell degeneration (Liang et al., 2012). Also, EP reduction is not a determined factor for deafness (Chen et al., 2014). However, it has been found that deletion of Cx26 before P4–5 can induce cochlear developmental disorders with congenital deafness, while deletion of Cx26 after P4–5 produces neither congenital deafness nor cochlear developmental disorders (Chen et al., 2014). These data strongly indicate that the congenital deafness is associated with cochlear developmental disorders. It has been found that deletion of Cx26 before P4–5 can induce the tectorial membrane attached at the inner sulcus cells leading to loss of the under-tectorial-membrane space; the cochlear tunnel is also filled (Wang et al., 2009; Liang et al., 2012; Chen et al., 2014). These developmental disorders can cause the tectorial membrane becoming immovable and are unable to stimulate hair cell transduction channels to produce auditory receptor current during acoustic stimulation (Liang et al., 2012), thereby leading to hearing loss.

Mouse models also showed that late-onset hearing loss caused by Cx26 deficiency was not associated with hair cell degeneration (Zhu et al., 2015a). Conditional KO (cKO) of Cx26 in the cochlea after birth could produce late-onset hearing loss. However, Cx26 cKO mice had no hair cell loss (Zhu et al., 2015a). It has been found that progressive, late-onset hearing loss is associated with the progressive reduction of outer hair cell (OHC) electromotility and active cochlear amplification (Zhu et al., 2013, 2015a), although there are neither connexin expression nor gap junctions in hair cells (Kikuchi et al., 1995; Zhao and Santos-Sacchi, 1999; Zhao and Yu, 2006; Yu and Zhao, 2009). These data further indicate that the K^+^-recycling defect hypothesis is not responsible for Cx26 deficiency induced late-onset hearing loss.

### Mechanisms Underlying Cx26 Deficiency Induced Cochlear Developmental Disorders and Gap Junction-Mediated miRNA Intercellular Transfer and Communication

Currently, the detailed mechanisms underlying Cx26 deficiency inducing cochlear developmental disorders are still unclear. However, recent experiments demonstrated that Cx26 deficiency-induced cochlear developmental disorders may result from the disruption of permeability of inner ear gap junctions to miRNAs (Zhu et al., 2015b).

Cx26 deficiency can cause disorders in the cochlear development (Wang et al., 2009; Liang et al., 2012; Chen et al., 2014), indicating that gap junction-mediated intercellular communication has a crucial role in cochlear development. Organ development relies on well-orchestrated intercellular communication to coordinate gene expression. Gene expression can be regulated by many factors at many stages, such as cis-element enhancers and promoters, epigenetic modifications such as chromatin modification and DNA methylation, trans-element transcription factors and non-coding RNAs at post-transcriptional level miRNA and mRNA polyA polymerization. However, except small non-coding regulatory RNAs, none of these regulators undergo intercellular exchange through gap junctional channels.

MicroRNAs (miRNAs) are small non-coding regulatory RNAs and ~20–25 nucleotides long (Bartel, 2004), forming a linear molecule with a diameter of <1.0 nm which is on the same order as the gap junction channel pore size (Brink et al., 2012). miRNAs play an important role in many physiological and pathological processes, including organ development. It has been found that deficiency of miRNAs can cause cochlear development disorders (Soukup et al., 2009; Conte et al., 2013). Cx26 deficiency induced cochlear developmental disorders are also found to be associated with the disruption of miRNA intercellular communication. It has been found that Cx26 KO disrupts permeability of inner ear gap junctions to miRNAs and the cochlea has developmental disorders, whereas Cx30 KO does not influence miRNA permeability and the cochlea displays normal development (Zhu et al., 2015b). Moreover, deafness mutation of Cx26 p.R75W can disrupt miRNA permeability (Zong et al., 2016), and Cx26 p.R75W transgenic mice also display cochlear developmental disorders and the cochlear tunnel is filled (Kudo et al., 2003). In addition, it has been found that miRNA expression is associated with gap junctional coupling. miR-96 has a critical role in the cochlear development (Conte et al., 2013). It has been found that miR-96 expression in the cochlea has a rapid increase at P2–3 just before the cochlear tunnel opening (Zhu et al., 2015b). However, this increasing peak is absent in Cx26 KO mice (Zhu et al., 2015b) in which the cochlear tunnel is filled (Chen et al., 2014; Zhu et al., 2015b). In Cx30 KO mice, in which the cochlear tunnel is developed normally (Teubner et al., 2003; Zhu et al., 2015b), the peak remains (Zhu et al., 2015b). These data indicate that Cx26 deficiency-induced cochlear developmental disorders may be associated with the disruption of permeability of inner ear gap junctions to miRNAs (Zhu et al., 2015b). However, the detailed underlying mechanism is still unclear. These data also demonstrate that the gap junction-mediated miRNA intercellular communication may have an important role in initiation and synchronization of miRNA expression among cells.

Previous studies demonstrated that miRNAs can be exchanged between cells in a gap junction-dependent manner (Valiunas et al., 2005, 2015; Kizana et al., 2009; Katakowski et al., 2010; Gregory et al., 2011; Lim et al., 2011; van Rooij et al., 2012; Hong et al., 2015; Suzhi et al., 2015; Menachem et al., 2016) and play a crucial role in the tumor generation and progression (Aasen et al., 2016). Our recent study further demonstrated that miRNAs can pass through gap junctional channels and regulate gene expression in neighboring cells to achieve an intercellular genetic communication (Zong et al., 2016). Moreover, we found that this gap junction-mediated miRNA intercellular gene regulation is connexin-dependent. For example, consistent with Cx30 channels impermeable to negative-charged molecules (Manthey et al., 2001; Beltramello et al., 2003), Cx30 channels are impermeable to miRNAs (Zong et al., 2016), since miRNAs appear anionic in physiological pH. Cx26 and Cx30 are predominant connexin isoforms in the cochlea (Forge et al., 2003; Zhao and Yu, 2006). Cx26 gap junctional channels are permeable to both anionic and cationic molecules, and are mainly responsible for permeability to anionic molecules in the cochlea (Zhao, 2005). Thus, Cx26 deficiency can affect passage of miRNAs in the cochlea, whereas Cx30 KO has no effect (Zhu et al., 2015b). This is consistent with finding that Cx26 KO but not Cx30 KO produces cochlear developmental disorders (Liang et al., 2012; Chen et al., 2014; Zhu et al., 2015b).

It has been reported that Cx26 expression in Cx30 KO mice is also reduced but not abolished (Ahmad et al., 2007; Ortolano et al., 2008); compensation of Cx26 expression can restore hearing function in Cx30 KO mice (Ahmad et al., 2007). These data further indicate that the Cx26 channels in the cochlea can be efficient to achieve permeability to anionic molecules, including miRNAs. These data also indicate that deafness in Cx30 KO mice resulted from differing underlying mechanisms as both Cx26 and Cx30 are reduced. Cx30 can assemble heterozygous (heterotypic/heteromeric) gap junctional channels with Cx26 in the cochlea (Zhao and Santos-Sacchi, 2000). Also, gap junctions formed in the Cx26/30 co-expressed cell line showed good permeability to fluorescent dyes (Yum et al., 2010; Zong et al., 2016) and miRNAs (Zong et al., 2016). It seems that there is no significant difference in permeation of Cx30, Cx26 and Cx26/30 heterozygous channels to K^+^ ions (Figure 1B).

Our recent study also demonstrated that the gap junction-mediated miRNA intercellular gene regulation is not specific to miRNAs (Zong et al., 2016). This is consistent with the fact that all miRNAs have a uniform structure and similar size (Bartel, 2004). Therefore, gap junctions can mediate all of miRNAs expression among cells and Cx26 deficiency can disrupt all of miRNAs intercellular transfer and cell-to-cell communication in the cochlea. Furthermore, considering one miRNA can affect the stability of hundreds of mRNAs and silence multiple genes (Bartel, 2004, 2009), thus, Cx26 deficiency can produce a broad effect and affect many gene expressions and regulations, which undoubtedly will affect cochlear development. However, the cochlear development is a complex process, involving coordination of many gene expressions and regulations. So far, the detailed mechanisms about how disruption of miRNA intercellular communication by Cx26 deficiency affects gene regulations to influence cochlear development still remain unclear.

### Summary

The K^+^-recycling defect hypothesis states that Cx26 deficiency may impair inner ear gap junctions and disrupt the gap junction-mediated K^+^-recycling pathway in the cochlea, thereby causing K^+^-ion accumulation around hair cells and toxicity, which eventually leads to hair cell degeneration and hearing loss. However, recent studies demonstrate that deletion of Cx26 can still retain inner ear gap junction permeability to ions, since co-expressed Cx30 exists. Hearing loss in Cx26 deficient mice also occurs in advance of hair cell degeneration or even without hair cell loss. Finally, the first step K^+^-sinking for K^+^-recycling in the cochlea is associated with ATP-purinergic P2X receptor activity rather than Cx26 expression. These data strongly suggest that K^+^ recycling hypothesis is not a Cx26 deficiency deafness mechanism, although the K^+^-recycling is important for normal hearing.

The fact that Cx26 deficiency can cause congenital deafness and late-onset hearing loss also demonstrates that they have different underlying deafness mechanisms rather than a unique deafness mechanism as assumed by K^+^-recycling defect hypothesis. Using different Cx26 deficient mouse models, it has been found that the congenital deafness induced by Cx26 deficiency is mainly resulted from cochlear developmental disorders, whereas the late-onset hearing loss is associated with the reduction of active cochlear amplification (Zhu et al., 2013, 2015a). Recent experiments further demonstrated that cochlear developmental disorders induced by Cx26 deficiency may be associated with disruption of gap junction-mediated miRNA intercellular communication for gene regulation in the cochlea. Advances in ATP-P2X purinergic receptor-mediated K^+^-sinking and gap junction-mediated miRNA intercellular gene regulation are also benefit to studies in other systems. However, many detailed mechanisms and key points, such as how disruption of miRNA intercellular transfer by Cx26 deficiency affects cochlear development, how miRNA intercellular transfer triggers and synchronizes miRNA expression among cells, and detailed mechanisms underlying ATP-purinergic receptor-mediated K^+^-sinking, still remain unclear and need to be further investigated in future.

## Author Contributions

H-BZ wrote the article.

## Conflict of Interest Statement

The author declares that the research was conducted in the absence of any commercial or financial relationships that could be construed as a potential conflict of interest.
